# Nutritional impact of CFTR modulators in children with cystic fibrosis

**DOI:** 10.3389/fped.2023.1130790

**Published:** 2023-03-29

**Authors:** Margaux Gaschignard, Fabien Beaufils, Florian Lussac-Sorton, Pauline Gallet, Haude Clouzeau, Joris Menard, Aurélie Costanzo, Lucie Nouard, Laurence Delhaes, Candice Tetard, Thierry Lamireau, Michael Fayon, Stéphanie Bui, Raphaël Enaud

**Affiliations:** ^1^ Bordeaux University Hospital, Hôpital Pellegrin-Enfants, Paediatric Cystic Fibrosis Reference Center (CRCM), Centre d'Investigation Clinique (CIC 1401), Bordeaux, France; ^2^ Centre de Recherche Cardio-Thoracique de Bordeaux, INSERM U1045, Bordeaux University, Bordeaux, France

**Keywords:** cystic fibrosis, nutrition, nutritional intake, macronutrient, vitamin E, selenium, lumacaftor, ivacaftor

## Abstract

**Background:**

Nutritional status is a major prognostic factor for breathing and the survival of patients with cystic fibrosis (CF). Since 2012, the development of CFTR modulators has considerably transformed the outcome of this disease. Indeed, both lung function and body mass index are improved by CFTR modulators, such as Lumacaftor/Ivacaftor. However, few data exist regarding the outcome of nutritional intakes under Lumacaftor/Ivacaftor.

**Methods:**

We conducted a prospective single-center study in children with CF treated with Lumacaftor/Ivacaftor to evaluate their nutritional intake before and after treatment

**Results:**

Thirty-four children were included in this study, with a median age of 12.4 years [11.9; 14.7]. There was no significant improvement in weight, height or BMI. Patients' total energy intake was not significantly changed with Lumacaftor/Ivacaftor, while carbohydrate intakes decreased significantly. We found that blood levels of vitamin E and Selenium were significantly increased under Lumacaftor/Ivacaftor, without a significant increase in supplementation. In patients with a BMI Z-score < 0 at treatment initiation, there was a significant improvement in weight and BMI Z-score, while TEI and carbohydrate intakes were significantly lower.

**Conclusion:**

We showed that treatment with Lumacaftor/Ivacaftor improved the nutritional status of patients without necessarily being associated with an increase in nutritional intake. Although these data need to be confirmed in larger cohorts, they support the hypothesis that weight gain under modulators is multifactorial, and may be related to a decrease in energy expenditure or an improvement in absorption.

## Introduction

Cystic fibrosis (CF) is a genetic disorder that affects multiple organ systems, particularly the respiratory and digestive systems. It is caused by a mutation in the gene encoding the cystic fibrosis transmembrane conductance regulator (CFTR) protein, which is essential for controlling the movement of ions and water across epithelial cell membranes (1).

The nutritional status is a key determinant of health and well-being in individuals with cystic fibrosis (CF). Poor nutritional status is a common complication of CF and is associated with increased morbidity, mortality, and reduced quality of life. Factors that increase the risk of malnutrition in CF include the difficulty in the absorption and utilization of nutrients due to exocrine pancreatic insufficiency (EPI), increased energy expenditure due to chronic inflammation and lung infections, intestinal inflammation, and a decreased appetite due to gastrointestinal symptoms (2). In this context, the nutritional management of patients with cystic fibrosis is an essential element of follow-up (3, 4).

Joint international recommendations from the European Society for Clinical Nutrition and Metabolism (ESPEN), the European Society for Pediatric Gastroenterology Hepatology and Nutrition (ESPGHAN) and the European Cystic Fibrosis Society (ECFS), published in 2016, reiterate the main principles of nutritional management of patients with CF, in particular a balanced and high-calorie diet, which should cover 110% to 200% of basic energy requirements (5). Despite these recommendations and appropriate nutritional management, the prevalence of undernutrition in cystic fibrosis patients remains higher than in the general population (3, 4, 6, 7).

However, the management of cystic fibrosis has made significant progress in recent years with the emergence of CFTR modulators, which aim to partially restore the functionality of the CFTR protein (8). The Lumacaftor/Ivacaftor combination (ORKAMBI®) is among the first available modulators and has been available in France since 2015, initially it was authorized for homozygous F508del patients from the age of 12, since 2019 it can be used from the age of 2 (9). The Lumacaftor/Ivacaftor combination has demonstrated effectiveness in improving respiratory function, with an increase in forced expiratory volume in one second (FEV1) and a reduction in exacerbations, but also on quality of life and body mass index (BMI) (10).

The improvement in BMI is probably due to several factors, including a decrease in energy expenditure due to improved respiratory function and a reduction in exacerbations (11, 12), a decrease in intestinal inflammation (13) and possibly an improvement in pancreatic function as reported in rare cases (14). However, data on changes innutritional intake with Lumacaftor/Ivacaftor are currently limited. The aim of this study was therefore to evaluate changes of nutritional intake in children treated with Lumacaftor/Ivacaftor in real life.

## Methods

### Study design and population

This study was conducted prospectively at the Bordeaux University Hospital and included children under 18 years of age with CF (sweat test > 60 mmol/L) who were eligible for Lumacaftor/Ivacaftor treatment in routine care and having had an assessment at home of nutritional intake before and after Lumacaftor/Ivacaftor initiation.

This was a non-interventional study as Lumacaftor/Ivacaftor has been approved by the Food and Drug Administration and the European Medicines Agency, and authorized in France since 2015 for F508del homozygous patients aged over 12 years, and from 2 years of age since 2019. Thus, at the time of the patient follow-up period, Lumacaftor/Ivacaftor was prescribed as part of the patients' routine care. Nutritional surveys were also performed as part of routine care. In addition, the use of the data collected and analyzed for the study were exclusively extracted from the medical records of the patients (MUCODOMEOS, https://www.vaincrelamuco.org/2019/05/09/mucodomeos-un-logiciel-adapte-aux-besoins-des-crcm-2684) after having obtained their informed consent. In this context and according to the French law in force, the approval of an ethics committee was not required.

Patients were divided into three age groups: 2–6 years, 7–11 years and over 12 years. The three age groups were defined according to the current ESPEN- ESPGHAN-ECFS recommendations and the current marketing authorization for Lumacaftor/Ivacaftor.

### Assessment of nutritional intake

In addition to the systematic evaluations conducted by the dieticians of the CF unit at each follow-up visit, an assessment of nutritional intake could be performed as part of the routine care in children with CF followed at the University Hospital of Bordeaux. These assessments were conducted at home, in patients with a stable condition and at least three months after an exacerbation, during three consecutive days (including one weekday and two weekend days). These assessments were set up at home by the dietitians of the patients' care provider, which means that patients whose health status did not require a care provider could not participate in this study.

This nutritional assessment consisted of a detailed collection of all daily meals and snacks as well as the various nutritional supplements and medications taken. Parents reported the quantities consumed, either by weighing the food or in household units. The content of food in protein, lipid, carbohydrates, micronutrients and vitamins was then calculated according to the CIQUAL 2012 food composition table (https://ciqual.anses.fr). The nutritional assessment was then sent to the dieticians of the CF department of Bordeaux University Hospital for analysis and comparison with the recommendations.

Macronutrient intakes were expressed in grams per kilogram per day (g/kg/d), kcal per kilogram per day (kcal/kg/day) and as a percentage of the recommended total energy intake (TEI). The TEI was the sum of spontaneous food intake and any nutritional support (oral nutritional supplements (ONS) or enteral nutrition (EN)).

Compliance with the recommendations was defined by: TIE of between 110% and 200% of the recommended dietary allowances in the general population for age, gender and an average physical activity level, of which 45% in the form of carbohydrates, 35% in the form of fat and 20% in the form of protein (5, 15). We then retained as recommended, the nutritional intakes in our series of cystic fibrosis patients:

– TEI > 100 kcal/kg/day between two and six years of age (90 kcal/kg/day recommended for the general population in this age group), including intakes of at least 5 g/kg/d of protein, 3.9 g/kg/d of fat and 11.3 g/kg/d of carbohydrates.
– TEI > 83 kcal/kg/day between seven and eleven years of age (75 kcal/kg/day recommended for the general population in this age group), with intakes of at least 4.2 g/kg/d of protein, 3.2 g/kg/d of fat and 9.3 g/kg/d of carbohydrates.
– TEI > 67 kcal/kg/day from age 12 onwards (60 kcal/kg/day recommended for the general population in this age group), including intakes of at least 3.3 g/kg/d of protein, 2.6 g/kg/d of fat and 7.5 g/kg/d of carbohydrates.

The nutritional assessments considered in this study were the last one performed before Lumacaftor/Ivacator initiation and the first one performed after treatment initiation.

### Collection of clinical and biological data

Demographic and cystic fibrosis-related data collected from the medical records of the patients were: age, sex, CFTR gene mutations, co-morbidities (EPI, neonatal ileus, liver disease, carbohydrate intolerance and diabetes, chronic colonization with *Staphylococcus aureus* or *Pseudomonas aeruginosa*), number of intravenous (IV) antibiotic courses in the last 12 months prescribed, treatments (in particular, vitamin supplementation), anthropometric data [weight, height and body mass index (BMI) expressed as Z-Score], patients' respiratory function [assessed by the percentage of predicted forced expiratory volume in 1 s (ppFEV1)], and blood levels of vitamins and oligoelements.

Clinical and biological data were obtained from annual check-ups performed in routine care. Nutritional assessments were performed approximately 3 months after the annual check-up.

### Statistical analyses

Analyses were only performed on available data using R software® (version 4.0). Parametric variables were compared using the Mann–Whitney test or ANOVA, and results were expressed as mean ± standard deviation (SD). Non-parametric variables were compared with the Mann–Whitney test for unpaired values or the Wilcoxon test for paired values and with the t-test for parametric variables. Results were expressed as the mean ± standard deviation (SD) or as the median and interquartile range [IQR]. Categorical variables were expressed as absolute values and percentages. Comparisons of categorical variables were analysed using the Fisher's exact test or Chi square test. Correlations were performed using the Spearman test. A *p*-value < 0.05 was considered significant.

## Results

### Description of the population

Among the 192 children with CF, followed at the University Hospital of Bordeaux, 63 were treated with Lumacaftor/Ivacaftor during the study period. One patient did not have a nutritional assessment prior to the initiation of Lumacaftor/Ivacaftor and 28 others (45%) did not have a nutritional assessment after the initiation of treatment. Consequently, 34 (54%) patients were included in this study.

Patient characteristics at baseline are summarized in Table 1. The median age before initiation of Lumacaftor/Ivacaftor treatment was 12.4 years [11.9; 14.7] and 18 patients (53%) were female. The median ppFEV1 at baseline was 77% [64%; 90%] and 32% of patients had chronic *P. aeruginosa* colonization. Concerning nutrition, the median Z-score for BMI was −0.5 [−1.2; 0.2]. Twenty-three patients (67%) had a BMI Z-score < 0, and 10 patients (29%) had a BMI Z-score < −1 at baseline. Twenty patients (59%%) received nutritional support (ONS or NE). All patients in our cohort had EPI at inclusion.

**Table 1:** Clinical characteristics at baseline and after lumacaftor/ivacaftor initiation (*n* = 34).

	Before initiation of Lumacaftor/ivacaftor	After initiation of Lumacaftor/ivacaftor	*p*-value
Female	18 (53%)	–	–
Age (years)	12.4 [11.9; 14.7]	13.2 [12.4; 15.8]	0.03
ppFEV1	77 [64; 90]	84.5 [73; 97]	0.13
Chronic *Staphylococcus aureus* colonisation	26 (76%)	26 (76%)	–
Chronic *Pseudomonas aeruginosa* colonisation	11 (32%)	11 (32%)	–
Number of IV antibiotic courses^*^	2 [1; 3]	1 [0; 2]	<0.001
Z-Score Weight	−0.5 [−1.2; 0.2]	−0.3 [−0.9; 0.5]	0.11
Z-Score Height	−0.4 [−1.4; 0.4]	−0.4 [−1.2; 0.5]	0.77
Z-Score BMI	−0.5 [−1.2; 0.2]	−0.38 [−0.7; 0.5]	0.2
**Nutritional support**			
ONS	14 (41%)	14 (41%)	–
EN	6 (18%)	6 (18%)	–
EPI	34 (100%)	34 (100%)	–
Pancreatic enzymes (U/kg/d)	6,250 [5,308; 7,668]	5,925 [4,878; 7,176]	0.19
Carbohydrate intolerance	22 (62%)	21 (61%)	–
Diabetes	6 (18%)	6 (18%)	–
Liver disease	7 (21%)	7 (21%)	–
Neonatal ileus	6 (18%)	–	–

Data are expressed as *n* (%) or median [IQR].

CFTR, cystic fibrosis transmembrane conductance regulator; EN, enteral nutrition; EPI, exocrine *p*ancreatic insufficiency; IV, intravenous; ONC, oral nutritional supplements; ppFEV1, percentage of predicted forced expiratory volume in 1.

^*^ In the last 12 months.

### Changes in clinical and nutritional outcomes with lumacaftor/ivacaftor

The median time to re-evaluation after the initiation of Lumacaftor/Ivacaftor was 10 months [6; 12]. Respiratory function did not significantly improve with Lumacaftor/Ivacaftor (median ppFEV1 before 77% [64; 90] vs. 84.5% [73; 97] after introduction of treatment, *p* = 0.13), but patients had a significant decrease in the number of IV antibiotic courses (2 [1; 3] vs. 1 [0; 2] respectively, *p* < 0.001) (Table 1).

Lumacaftor/ivacaftor did not significantly improve weight, height or BMI Z-score (Table 1). Changes innutritional intakes with Lumacaftor/Ivacaftor are shown in Table 2. Patients' TEI did not significantly change after Lumacaftor/Ivacaftor initiation, while carbohydrate intakes significantly decreased (median 8.9 g/kg/d [7.3; 11.1] before vs. 7.8 g/kg/d [5.2; 9.4] after treatment initiation, *p* = 0.01). Lipid and protein intakes were not significantly affected. Compliances with nutritional recommendations before and after treatment initiation is shown in Table 3*.* There was no significant difference under treatment. Nutritional support was unchanged with Lumacaftor/Ivacaftor in the 14 children (41%) who received it at baseline. There was no correlation between changes in TEI and anthropometric parameters (weight, height, or BMI Z-score) or respiratory function under treatment. However, we observed a negative correlation between baseline TEI and improvement in TEI after the introduction of treatment (r = −0.3; *p* = 0.05).

**Table 2:** Changes in macronutrient intakes with lumacaftor/ivacaftor.

	Before initiation	After initiation	*p*-value
TEI (Kcal/kg/d)	86.4 [65.8; 102]	76.4 [58.1; 104]	0.08
Carbohydrate (g/kg/d)	8.9 [7.3; 11.1]	7.8 [5.2; 9.4]	0.01
Lipid (g/kg/d)	3.1 [2.2; 3.7]	3 [2.2; 3.7]	0.59
Protein (g/kg/d)	2.9 [2.4; 3.5]	2.9 [2.5; 3.4]	0.41

Data are expressed as median [IQR]. TEI, total energy intake.

**Table 3:** Changes in compliance with nutritional recommendations after initiation of lumacaftor/ivacaftor.

	Before initiation	After initiation	*p*-value
TEI	20 (59%)	21 (62%)	0.80
Carbohydrate intake	22 (65%)	19 (56%)	0.46
Lipid intake	16 (47%)	21 (62%)	0.22
Protein intake	9 (34%)	10 (29%)	1

Data are expressed as *n* (%). TEI, Total energy intake.

Changes in supplementation, blood levels of vitamins and micronutrients is shown in Table 4*.* We found that blood levels of vitamin E were significantly increased under treatment, while the daily dose of vitamin E supplementation was significantly lower. In addition, blood selenium levels were significantly increased without treatment, with no significant increase in supplementation. There were no significant changes in blood levels and supplementation for other vitamins and micronutrients.

**Table 4:** Changes in supplementation and blood levels of vitamins and micronutrients with lumacaftor/ivacaftor.

	Before initiation	After initiation	*p*-value
**Vitamin A**			
Dose (U/kg/)	489 [192; 1144]	541 [172; 1053]	0.75
Blood rate (mg/L)	1.19 [0.96; 1.46]	1.35 [1.14; 1.64]	0.2
**Vitamin D**			
Dose (U/kg/j)	168 [110; 240]	195 [128; 292]	0.73
Blood rate (ng/ml)	31.2 [28.6; 39]	26.9 [21.8; 35.7]	0.16
**Vitamin E**			
Dose (U/kg/j)	14.5 [8; 19.5]	12.5 [8; 16]	<0.01
Blood rate (mg/L)	16.4 [13.9; 20.2]	20.9 [16.9; 24.9]	<0.01
**Vitamin K**			
Monthly dose (mg)	40 [20; 40]	40 [20; 40]	0.6
Blood rate (mg/L)	103 [55.0; 222]	90.0 [49.0; 163	0.5
**Zinc**			
Dose Zinc	5.00 [5.00; 5.00]	5.00 [5.00; 5.00]	1
Blood rate (µmol/L)	13.7 [12.3; 15.9]	14.1 [13.6; 14.9]	0.97
**Selenium**			
Dose Selenium (/d)	100 [100; 100]	100 [100; 100]	1
Blood rate (µmol/L)	0.96 [0.86; 1.09]	1.08 [0.97; 1.19]	<0.001

Data are expressed as median [IQR].

### Changes in nutritional status and intake under lumacaftor/ivacaftor in patients with a BMI < 0 at treatment initiation

**Figure 1:**
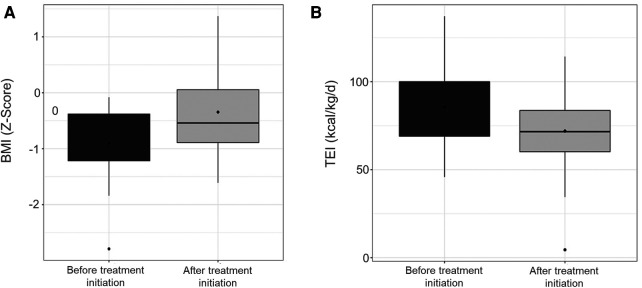
Z-score changes in body mass index (**A**) and total energy intake (**B**) under lumacaftor/ivacaftor.

We performed a subgroup study according to BMI at Lumacaftor/Ivacaftor initiation to assess whether baseline nutritional status influenced changes in nutritional intakes with Lumacaftor/Ivacaftor. Twenty-three patients (68%) had a BMI Z-score < 0 at Lumacaftor/Ivacaftor initiation. The median time to re-evaluation after initiation of Lumacaftor/Ivacaftor in this subgroup was 10 months [6.5; 12]. Under treatment, there was a significant improvement in weight and BMI Z-score, while TEI and carbohydrate intakes were significantly lower (Table 5 and Figure 1). ppFEV1 increased after Lumacaftor/Ivacaftor but not significantly, while the number of IV antibiotic courses decreased significantly under treatment.

**Table 5:** Changes in clinical data and macronutrient intakes with lumacaftor/ivacaftor in patients with BMI < 0 at baseline.

	Before initiation	After initiation	*p*-value
ppFEV1	77 [62.0; 84.0]	81.0 [70.5; 94.0]	0.12
Number of IV antibiotic courses^*^	2 [1; 3]	1 [0; 1]	<0.001
Z-Score Weight	−0.9 [−1.5; −0.3]	−0.3 [−1.0; 0.2]	0.04
Z-Score Height	−1 [−1.5; 0.3]	−0.4 [−1.4; 0.45]	0.52
Z-Score BMI	−0.8 [−1.2; −0.4]	−0.5 [−0.9; 0.05]	<0.01
TEI (Kcal/kg/d)	81 [69; 100]	72 [60; 84]	0.03
Carbohydrate (g/kg/d)	10.0 [8.7; 11.8]	8.3 [6.3; 9.7]	<0.01
Lipid (g/kg/d)	3.3 [2.5; 4.0]	3.1 [2.4; 3.7]	0.43
Protein (g/kg/d)	3.1 [2.7; 3.67]	3.0 [2.5; 4.3]	0.72

Data are expressed as median [IQR].

IV, intravenous; TEI, total energy intake.

^*^ In the last 12 months.

For the remaining 11 patients with a BMI Z-score > 0 at Lumacaftor/Ivacaftor initiation, there were no significant changes in anthropometric data and macronutrient intakes whilst under Lumacaftor/Ivacaftor.

## Discussion

Nutritional status is a major prognostic factor of the respiratory and survival of patients with CF. Nutritional management is therefore essential in the multidisciplinary management of these patients. The aim of this study was to evaluate nutritional intakes before and after the initiation ofLumacaftor/Ivacaftor in children with CF. Although we did not observe a significant improvement in anthropometric data (weight, height and BMI) among the 34 patients included, this study provides data on nutritional intake under Lumacaftor/Ivacaftor. Whilst on treatment, TEI tended to decrease (86 kcal/kg/d [66; 102] before vs. 76 [58; 102] after initiation, *p* = 0.08), without any impact on weight or BMI. Carbohydrate intakes were significantly reduced (median 8.9 g/kg/d [7.3; 11.1] before vs. 7.8 g/kg/d [5.2; 9.4] after treatment, *p* = 0.01), whereas protein and lipid intakes did not significantly change. Data in the literature regarding nutritional changes under CFTR modulators is scarce. A study conducted on American and Italian patients treated with Ivacaftor showed that the American participants significantly increased their lipid intake, whereas the Italian participants increased both their TEI and lipid intakes under treatment (16). These results may be due in part to the instructions concerning drug administration, indeed it is advised to take it whist eating food containing lipids.

In patients with a BMI Z-score < 0 at treatment initiation, we could show a significant improvement in weight and BMI after treatment initiation, while TEI and carbohydrate intakes were significantly decreased. Few studies have explored the mechanisms of weight gain under CFTR modulators, but the hypotheses may be increased caloric intake (which is not the case here), but it also reduces resting energy expenditure and improves intestinal absorption.

In contrast to the symptomatic therapeutic approaches still used in CF, therapeutic modulation of CFTR offers the potential for early intervention in younger and younger patients who may have preserved lung function and normal growth. The gastrointestinal tract then offers the possibility of new therapeutic targets for assessing the efficacy of CFTR modulators. The main objective of this study was to evaluate possible changes in dietary intake under treatment to try to explain the improvement in nutritional status with modulators, but other issues should be explored such as changes in intestinal transit time, intestinal pH, intestinal absorption of bile salts, exocrine pancreatic function, intestinal lipid malabsorption or intestinal inflammation (17).

Tétard et al. (13) demonstrated that faecal calprotectin, a biomarker of intestinal inflammation, was decreased on Lumacaftor/Ivacaftor. Furthermore, Dhaliwal et al. (18) showed in cystic fibrosis patients that intestinal inflammation was associated with poorer nutritional status, particularly in terms of increased energy expenditure and reduced intestinal absorption of fat-soluble vitamins (19). Reducing intestinal inflammation could therefore improve the nutritional status of patients. However, we did not have the data and biomarkers to assess changes in intestinal inflammation in these patients.

Although no patients in our study restored their pancreatic function whilst on treatment, and no significant changes in pancreatic enzyme supplementation were given, improvements in pancreatic function in patients treated with Ivacaftor or Lumacaftor/Ivacaftor have been described, and may also contribute to improved nutritional status in patients on treatment (14, 20, 21). An improvement, even partial, of the exocrine pancreatic function could also explain the increase in blood levels of vitamin E observed under treatment. The evolution of pancreatic function with CFTR modulators is therefore an essential element to investigate in the nutritional changes with CFTR modulators, especially in populations initiating treatment at two years of age.

In terms of vitamins and micronutrients, we found that blood levels of Vitamin E were significantly increased after treatment initiation, as previously observed, while Vitamin E supplementation was significantly decreased (22). Gelzo et al. evaluated sterol metabolism and intestinal cholesterol absorption under Lumacaftor/Ivacaftor (22). Vitamin E and cholesterol levels were significantly increased after the introduction of the treatment, indicating an improvement in digestive absorption of lipids. The improvement in intestinal absorption may be explained by the efficacy of Lumacaftor/Ivacaftor on cystic fibrosis digestive damage. An other explanation may be that Lumacaftor is a known inducer of cytochrome P450 3A4 and may also affect the breakdown of vitamin E (18). Another hypothesis is that the risk of vitamin E deficiency increases with inflammation of the respiratory or digestive system (23, 24) and Lumacaftor/Ivacaftor has been shown to decrease pulmonary and digestive inflammation (13, 25).

Blood levels of selenium also increased significantly without any change in supplementation. Anti-inflammatory and antioxidant properties have been described with Selenium. One of the hypotheses that we could propose would be an increase in blood selenium levels associated with a decrease in intestinal inflammation under modulators, either with a decrease in consumption and/or an increase in absorption, as described in patients with inflammatory bowel disease in remission (26). There was no significant change in the supplementations and blood levels of other vitamins and trace elements, whereas an increase in Vitamin D intake and Vitamin A plasma levels has been described with Ivacaftor (27).

However, our study has some limitations. The nutritional assessments were based on a dietary survey completed at home by the patient and his/her parents, which may lead to a bias in data collection. Furthermore, we could not show a significant improvement in BMI in our cohort, although this is one of the most consistent criteria in the literature (8, 23, 27). These results may be explained by a lack of power in our cohort. We did not observe a significant difference in ppFEV1 with treatment. Although real-life data showed a 3% increase in ppFEV1 after 6 months of treatment (23), the evolution of pulmonary function is multifactorial (and notably related to local inflammation or the presence of bronchiectasis), as shown by the partial correlation between changes in sweat test and FEV1 (24–26). However, we observed an improvement in intravenous antibiotic courses, which reflect more consistent effectiveness outcomes according to numerous studies (8, 25, 28–30). Finally, the median time to re-evaluation after Lumacaftor/Ivacaftor was 10 months [6; 12]. A real-life, long-term study (e.g., re-evaluation at 2 years) may be relevant in order to improve the detection of the effects of Lumacaftor/Ivacaftor treatment on nutritional status (31).

## Conclusion

This study provides data on nutritional intake in children with CF treated with Lumacaftor/Ivacaftor. Despite a limited impact on weight, BMI and TEI, we were able to observe an increase in carbohydrate intake under Lumacaftor/Ivacaftor. The nutritional benefits of this treatment appear to depend in part on the nutritional status at treatment initiation. Indeed, they appear to be greater in the patients with a BMI Z-score < 0, for whom we were able to demonstrate an improvement in weight and BMI, while they decreased their TEI and carbohydrate intake. An enhanced absorption of vitamin E and selenium also seems to be observed under treatment. Although these data need to be confirmed in larger cohorts, they support the hypothesis that weight gain under modulators is multifactorial, and may be related to a decrease in energy expenditure or an improvement in absorption. Nonetheless, these hypotheses need to be the subject of dedicated studies. It would also be interesting to study in parallel the parameters of intestinal inflammation and absorption to improve the knowledge of the mechanisms allowing a nutritional improvement under modulators.

If these results are confirmed, current nutritional recommendations for patients with CF will probably have to be adapted in patients on modulators in order to avoid long-term complications, in particular those related to the increased frequency of overweight and obesity, as we have already observed in patients treated with Ivacaftor (32).

## Data Availability

The raw data supporting the conclusions of this article will be made available by the authors, without undue reservation.

## References

[1] O'Sullivan BP Freedman SD. Cystic fibrosis. Lancet. (2009) 373:1891–904. 10.1016/S0140-6736(09)60327-5 19403164

[2] Brownell JN Bashaw H Stallings VA. Growth and nutrition in cystic fibrosis. Semin Respir Crit Care Med. (2019) 40:775–91. 10.1055/s-0039-1696726 31659726

[3] Borowitz D Durie PR Clarke LL Werlin SL Taylor CJ Semler J Gastrointestinal outcomes and confounders in cystic fibrosis. J Pediatr Gastroenterol Nutr. (2005) 41:273–85. 10.1097/01.mpg.0000178439.64675.8d 16131979

[4] Sinaasappel M Stern M Littlewood J Wolfe S Steinkamp G Heijerman HGM Nutrition in patients with cystic fibrosis: a European consensus. J Cyst Fibros. (2002) 1:51–75. 10.1016/S1569-1993(02)00032-2 15463811

[5] Turck D Braegger CP Colombo C Declercq D Morton A Pancheva R ESPEN-ESPGHAN-ECFS guidelines on nutrition care for infants, children, and adults with cystic fibrosis. Clin Nutr. (2016) 35:557–77. 10.1016/j.clnu.2016.03.004 27068495

[6] Culhane S George C Pearo B Spoede E. Malnutrition in cystic fibrosis. Nutr Clin Pract. (2013) 28:676–83. 10.1177/0884533613507086 24170579

[7] Filigno SS Robson SM Szczesniak RD Chamberlin LA Baker MA Sullivan SM Macronutrient intake in preschoolers with cystic fibrosis and the relationship between macronutrients and growth. J Cyst Fibros. (2017) 16:519–24. 10.1016/j.jcf.2017.01.010 28185886 PMC5494266

[8] Wainwright CE Elborn JS Ramsey BW Marigowda G Huang X Cipolli M Lumacaftor–ivacaftor in patients with cystic fibrosis homozygous for Phe508del *CFTR*. N Engl J Med. (2015) 373:220–31. 10.1056/NEJMoa1409547 25981758 PMC4764353

[9] Boyle MP Bell SC Konstan MW McColley SA Rowe SM Rietschel E A CFTR corrector (lumacaftor) and a CFTR potentiator (ivacaftor) for treatment of patients with cystic fibrosis who have a phe508del CFTR mutation: a phase 2 randomised controlled trial. Lancet Respir Med. (2014) 2:527–38. 10.1016/S2213-2600(14)70132-8 24973281

[10] Habib A-RR Kajbafzadeh M Desai S Yang CL Skolnik K Quon BS. A systematic review of the clinical efficacy and safety of CFTR modulators in cystic fibrosis. Sci Rep. (2019) 9:7234. 10.1038/s41598-019-43652-2 31076617 PMC6510767

[11] Konstan MW McKone EF Moss RB Marigowda G Tian S Waltz D Assessment of safety and efficacy of long-term treatment with combination lumacaftor and ivacaftor therapy in patients with cystic fibrosis homozygous for the F508del-CFTR mutation (PROGRESS): a phase 3, extension study. Lancet Respir Med. (2017) 5:107–18. 10.1016/S2213-2600(16)30427-1 28011037

[12] Southern KW Murphy J Sinha IP Nevitt SJ. Corrector therapies (with or without potentiators) for people with cystic fibrosis with class II CFTR gene variants (most commonly F508del). Cochrane Database Syst Rev. (2020) 12:CD010966. 10.1002/14651858.CD010966.pub3 33331662 PMC8094390

[13] Tétard C Mittaine M Bui S Beaufils F Maumus P Fayon M Reduced intestinal inflammation with lumacaftor/ivacaftor in adolescents with cystic fibrosis. J Pediatr Gastroenterol Nutr. (2020) 71:778–81. 10.1097/MPG.0000000000002864 32740537

[14] Crowley J Croinin K Mullane D Chróinín MN. Restoration of exocrine pancreatic function in child with lumacaftor/ivacaftor therapy in cystic fibrosis. J Cyst Fibros. (2022) 21:264. 10.1016/j.jcf.2021.08.032 34511391

[15] FAO/WHO/UNU Expert Consultation. Human energy requirements. Rome: World Health Organization (2004).

[16] Sainath NN Schall J Bertolaso C McAnlis C Stallings VA. Italian And north American dietary intake after ivacaftor treatment for cystic fibrosis gating mutations. J Cyst Fibros. (2019) 18:135–43. 10.1016/j.jcf.2018.06.004 29983355

[17] Bodewes FAJA Verkade HJ Taminiau JAJM Borowitz D Wilschanski M. Cystic fibrosis and the role of gastrointestinal outcome measures in the new era of therapeutic CFTR modulation. J Cyst Fibros. (2015) 14:169–77. 10.1016/j.jcf.2015.01.006 25677689

[18] Brigelius-Flohé R. Vitamin E and drug metabolism. Biochem Biophys Res Commun. (2003) 305:737–40. 10.1016/s0006-291x(03)00811-8 12763054

[19] Dhaliwal J Leach S Katz T Nahidi L Pang T Lee JM Intestinal inflammation and impact on growth in children with cystic fibrosis. J Pediatr Gastroenterol Nutr. (2015) 60:521–6. 10.1097/MPG.0000000000000683 25539196

[20] Munce D Lim M Akong K. Persistent recovery of pancreatic function in patients with cystic fibrosis after ivacaftor. Pediatr Pulmonol. (2020) 55:3381–3. 10.1002/ppul.25065 32910556

[21] Nichols AL Davies JC Jones D Carr SB. Restoration of exocrine pancreatic function in older children with cystic fibrosis on ivacaftor. Paediatr Respir Rev. (2020) 35:99–102. 10.1016/j.prrv.2020.04.003 32386958

[22] Gelzo M Iacotucci P Caputo M Cernera G Comegna M Carnovale V Lumacaftor/ivacaftor improves liver cholesterol metabolism but does not influence hypocholesterolemia in patients with cystic fibrosis. J Cyst Fibros. (2021) 20:e1–6. 10.1016/j.jcf.2020.06.015 32586737

[23] Brigelius-Flohé R. Vitamin E: the shrew waiting to be tamed. Free Radic Biol Med. (2009) 46:543–54. 10.1016/j.freeradbiomed.2008.12.007 19133328

[24] Hakim F Kerem E Rivlin J Bentur L Stankiewicz H Bdolach-Abram T Vitamins A and E and pulmonary exacerbations in patients with cystic fibrosis. J Pediatr Gastroenterol Nutr. (2007) 45:347–53. 10.1097/MPG.0b013e31804069e5 17873748

[25] Graeber SY Boutin S Wielpütz MO Joachim C Frey DL Wege S Effects of lumacaftor–ivacaftor on lung clearance Index, magnetic resonance imaging, and airway microbiome in Phe508del homozygous patients with cystic fibrosis. Ann Am Thorac Soc. (2021) 18:971–80. 10.1513/AnnalsATS.202008-1054OC 33600745

[26] Vaghari-Tabari M Jafari-Gharabaghlou D Sadeghsoltani F Hassanpour P Qujeq D Rashtchizadeh N Zinc and selenium in inflammatory bowel disease: trace elements with key roles? Biol Trace Elem Res. (2021) 199:3190–204. 10.1007/s12011-020-02444-w 33098076

[27] Sommerburg O Hämmerling S Schneider SP Okun J Langhans C-D Leutz-Schmidt P CFTR Modulator therapy with lumacaftor/ivacaftor alters plasma concentrations of lipid-soluble vitamins A and E in patients with cystic fibrosis. Antioxidants. (2021) 10:483. 10.3390/antiox10030483 33808590 PMC8003491

[28] Burgel P-R Munck A Durieu I Chiron R Mely L Prevotat A Real-life safety and effectiveness of lumacaftor-ivacaftor in patients with cystic fibrosis. Am J Respir Crit Care Med. (2020) 201:188–97. 10.1164/rccm.201906-1227OC 31601120

[29] Aalbers BL de Winter-de Groot KM Arets HGM Hofland RW de Kiviet AC van Oirschot-van de Ven MMM Clinical effect of lumacaftor/ivacaftor in F508del homozygous CF patients with FEV1 ≥ 90% predicted at baseline. J Cyst Fibros. (2020) 19:654–8. 10.1016/j.jcf.2019.12.015 31924546

[30] Taylor-Cousar JL Jain M Barto TL Haddad T Atkinson J Tian S Lumacaftor/ivacaftor in patients with cystic fibrosis and advanced lung disease homozygous for F508del-CFTR. J Cyst Fibros. (2018) 17:228–35. 10.1016/j.jcf.2017.09.012 29126871

[31] Bui S Masson A Enaud R Roditis L Dournes G Galode F Long-term outcomes in real life of lumacaftor-ivacaftor treatment in adolescents with cystic fibrosis. Front Pediatr. (2021) 9:744705. 10.3389/fped.2021.744705 34869102 PMC8634876

[32] Guimbellot JS Baines A Paynter A Heltshe SL VanDalfsen J Jain M Long term clinical effectiveness of ivacaftor in people with the G551D CFTR mutation. J Cyst Fibros. (2021) 20:213–9. 10.1016/j.jcf.2020.11.008 33249004 PMC8183611

